# Strategies for Selecting, Managing, and Engaging Undergraduate Coauthors: A Multi-Site Perspective

**DOI:** 10.3389/fpsyg.2019.00325

**Published:** 2019-02-26

**Authors:** Jenna L. Scisco, Jennifer A. McCabe, Albee Therese O. Mendoza, Marianne Fallon, Melanie M. Domenech Rodríguez

**Affiliations:** ^1^Department of Psychological Science, Eastern Connecticut State University, Willimantic, CT, United States; ^2^Center for Psychology, Goucher College, Baltimore, MD, United States; ^3^Department of Psychology, Wesley College, Dover, DE, United States; ^4^Department of Psychological Science, Central Connecticut State University, New Britain, CT, United States; ^5^Department of Psychology, Utah State University, Logan, UT, United States

**Keywords:** publishing, undergraduates, selection, management, engagement

In 2018, we delivered a symposium on publishing with undergraduate coauthors in the *Psi Chi Journal of Psychological Research* (Fallon, 2018a; Fallon and Domenech Rodríguez, 2018a,b; Fallon and Scisco, 2018; McCabe and Mendoza, 2018). Based on our collective experience, we identified three common challenges: effectively selecting, managing, and engaging students throughout the publication process. We use our perspectives from different institutions (i.e., small liberal arts colleges, mid-sized regional universities, and a large research university) and evidence from past research to provide strategies to successfully meet these challenges. Ultimately, the actionable strategies we describe could be used by a wide faculty readership to increase rates of successful publishing with undergraduate students.

## Selecting Undergraduate Coauthors

To maximize the chances of successful publication, it is desirable to select students whose academic and interpersonal qualities predict publishing success (1). Additionally, to increase diverse perspectives within psychology, faculty can recruit students from traditionally underrepresented groups (2).

1. Certain habits of mind may predict proactive behaviors (e.g., seeking feedback) needed to be successful undergraduate researchers (Eagan et al., 2011). Specifically, faculty should seek students who exhibit a growth mindset (Dweck, 2006): those who concern themselves with learning (vs. looking smart); who persist through challenges (vs. taking the easy path); who learn from criticism (vs. ignore or avoid it); and who believe effort (vs. innate intelligence) is the means to mastery. Sometimes the “smartest” students on paper may not embrace a growth mindset.
  Thus, mentors should consider potential student-collaborators from all levels and classes (Detweiler-Bedell et al., 2016). Though you are more likely to find someone (e.g., a student who has taken research methods) more prepared to engage in research writing in an advanced course, this overt training is not the only factor in evaluating potential. Keep an eye out for that special student–that diamond in the rough–who shows a curiosity about learning, dedication to academics, enthusiasm in “going the extra mile,” and an interpersonal style that meshes well with yours. If you are inclined to have potential research assistants complete an application as an initial screening, include not only their interest in working with you and their strengths/weaknesses as a researcher, but also their motivations for learning.
2. Non-first-generation students and those who identify as male are more likely to engage in undergraduate research compared to those who identify as female and first-generation students (Webber et al., 2013). Yet research suggests that ethnic minority students who are engaged in faculty-mentored research are more likely to be retained, persist in their studies, and academically succeed (Nagda et al., 1998; Jones et al., 2010).

Faculty can help make scientific research more inclusive by revealing the “hidden curriculum” of college. Some students arrive in college inherently recognizing the value of research collaboration and knowingly approach faculty about such opportunities. But many—especially first-generation college students and those from racial and ethnic minority groups—do not. Thus, faculty should make an overt effort with *all* students to clearly define collaborative research and emphasize its value for skill development in preparation for the workforce and/or graduate school (Bangera and Brownell, 2014).

One strategy is to introduce the idea of research collaboration or even assign an article on the value of student publication (see Anderson et al., 2015), even in introductory courses. If you have lab assistants or directed research students, have them discuss their experiences and their aspirations with the class. Although many students may not move forward, you may discover a potential coauthor who may have otherwise flown under the radar. Finally, take a retrospective and current look at the diversity of your undergraduate collaborators. Be aware of potential implicit bias in student-collaborator choice. Intentionally consider the students of color and whether they have the attributes discussed in point 1. Reach out to students who you suspect have this potential, even if they have not fully demonstrated it. Invite them to a conversation about what you do, why you love it, and how they can be involved. These are a few small but important steps toward equity and inclusion.

## Managing Undergraduate Coauthors

After students have been selected to write a manuscript as a coauthor, faculty should make a clear plan for publishing which includes: developing realistic timelines and expectations (1), identifying appropriate journals (2), discussing authorship order (3), and teaching students how to write publishable manuscripts (4).

1. Managing students' expectations about publishing may begin with an evaluation of the research topic to ensure that it is neither too difficult nor too trivial for publication. Managing expectations also begins with discussing and agreeing on a timeline of tasks as well as an outline of expectations in the progression of the manuscript. Roig (2007) and Cramblet Alvarez (2013) provide examples of student-faculty research and publication agreements which include weekly tasks and deadline dates, academic integrity policies, and specific guidelines on how tasks connect to authorship. This agreement could also explicitly articulate behaviors faculty expect to observe in student collaborators, including being honest about mistakes, asking questions well before deadlines, and responding to emails in a timely manner. In each step of the publication process, mentors may also consider incorporating learning exercises to make explicit the tasks needed to publish research (e.g., critically evaluating a journal article; Gottfried, 2009 or mastering APA style; Freimuth, 2008).
2. Currently, there are several psychology journals that specifically encourage and welcome submissions from undergraduate coauthors, including the *Psi Chi Journal of Psychological Research*, the *Journal of Psychology and Behavioral Sciences*, and the *Yale Review of Undergraduate Research in Psychology* (University of Nebraska-Lincoln (UNL) Libraries, 2018). If the research provides a unique contribution within a specific subfield, the faculty mentor may use her expertise to develop a list of appropriate outlets. Then, the faculty mentor and student can evaluate the submission and evaluation criteria for relevant journals as well as the timeline of publication to decide if the journal is a good fit for the project.
3. Clearly establishing author ordership and corresponding responsibilities at the start of the writing process can be very helpful for avoiding possible confusion and conflict. Some journals dictate that the lead author be an undergraduate or was an undergraduate when the research was conducted. As a guide, Fine and Kurdek (1993) propose ethical considerations and scenarios as well as practical recommendations to determine authorship between students and faculty mentors. For example, it may be helpful for both parties to engage in an informed consent process of sorts (written agreements recommended), in which the student is informed of the authorship decision-making process including the tasks necessary for publication (e.g., revising drafts before submission, reading submission guidelines), expectations for order of authorship (e.g., who completes what section, who addresses what revisions), and renegotiations of authorship depending on the amount of revision necessary (APA Science Student Council, 2006).
4. Although writing a research paper for a methods course and writing a research manuscript draw upon the same skills, undergraduates may be surprised at how challenging this transition can be. Before embarking on the writing, it may be helpful for students and faculty to read articles geared toward emergent researchers about writing empirical manuscripts in psychology (Fallon, 2018b). Detweiler-Bedell and Detweiler-Bedell's (2013) comprehensive guide for collaboratively writing manuscripts in APA style is particularly useful for addressing the challenges of group writing. It would also be helpful to review other manuscripts from the target journal or manuscripts from published undergraduates as exemplars. Checklists for each section of the manuscript can guide students through the writing process and keep them focused (Appelbaum et al., 2018). Faculty members may choose to focus on aspects of the manuscript requiring their expertise, such as locating high-fidelity citations, ensuring that effect sizes are included in statistical analyses, and justifying small sample sizes. Ultimately, the writing process will involve numerous rounds of revision, leading to the next challenge: keeping undergraduate coauthors engaged in the writing process.

## Engaging Undergraduate Coauthors

Students' time demands—coursework, internships, and employment—may compete with time dedicated to the writing process. Furthermore, given the lengthy nature of the publication process, students may graduate prior to manuscript publication. To engage undergraduate coauthors throughout the publication pipeline, we advocate providing timely communication and feedback on student work (1), offering regular encouragement and support (2), emphasizing the contribution to the field (3), and mentoring students in their response to reviewers (4).

1. At the beginning of the project, in addition to developing student timelines as described above, faculty members can establish guidelines for when they will respond to students' work. For example, faculty members may indicate that they will respond to emails within 24 h during the work week, and that they will provide feedback on manuscript drafts within 1 week. Following through on these communication and feedback guidelines can keep the project moving forward and continually engage the student. Weekly meetings with the student can be exceptionally helpful because they provide an opportunity to ask questions, discuss feedback, develop a positive working relationship, and keep both faculty and students on track. If students have graduated and are living nearby, face-to-face meetings could be continued. However, if on-campus meetings are not feasible, meetings can be held using video conferencing. Programs that allow for screen sharing (e.g., Skype, Zoom, GoogleDocs) are particularly helpful for simultaneously viewing parts of the manuscript.
2. First-time undergraduate coauthors may find faculty mentors' extensive, ongoing feedback and editing overwhelming or discouraging. To keep the student engaged, feedback should be positive and instructive. For example, if students struggle with integration of sources for a literature review, faculty members might say: “I see you have worked really hard to find relevant sources and describe each one. The next step is to tie these studies together into one paragraph around a common theme. Here is an example of how to start with a strong topic sentence and use the literature to develop that topic.” Such feedback helps students learn how to improve their writing and implicitly conveys that students can reach this goal, thereby increasing their writing motivation (Truax, 2018).
3. For undergraduate psychology students, benefits of co-writing and publishing research may include improved critical thinking and investigative skills (Beckman and Hensel, 2009) and increased confidence and interest to further produce publishable research (Griffiths, 2015). Benefits to the scientific community and broader society may include the dissemination of new knowledge, replication of previous findings, support for existing theories, ideas for future research, or practical implications of the findings. Faculty could tie these personal and broader benefits to students' futures. For example, if students aim to apply to graduate school, a published manuscript can provide a competitive advantage (Hartley, 2014). Students who plan to enter the job market can leverage the skills in communication, self-assessment, project management, and collaboration.
4. After providing clear communication, giving supportive feedback, and motivating students to continue the writing process, the completed manuscript will be submitted, and many students will feel as if they have reached the end of a long journey. However, the excitement of submission may be tempered by receiving many comments from reviewers and editors. To address students' potential deflation, faculty can share reviewer responses from other published works, demonstrating that multiple reviewer suggestions are a normal part of the publication process. Faculty can also model ways to appropriately respond to reviewers' comments including thanking the reviewer for their time and effort, acknowledging and changing unclear elements of the paper, and addressing each reviewer comment with an individual response (Guyatt and Brian Haynes, 2006). Further, faculty mentors play a critical role in helping students decide when they should respectfully disagree with reviewer suggestions. Sharing previous response letters and revisions that resulted in successful publication will give students a model to follow.

## Conclusion

Publishing with undergraduate coauthors may introduce unique challenges for faculty mentors, but employing the strategies we have described can make the publication process manageable, enjoyable, and successful. With a clear and thorough plan, faculty mentors will not only help students meaningfully contribute to our science, but will also prepare a new generation of scientifically literate and skilled young adults.

## Author Contributions

JS, JM, AM, MF, and MD conceptualized and revised the manuscript. JS, JM, and AM wrote the first draft of the manuscript.

### Conflict of Interest Statement

The authors declare that the research was conducted in the absence of any commercial or financial relationships that could be construed as a potential conflict of interest.
